# Gene modification therapies for hereditary diseases in the fetus

**DOI:** 10.1002/pd.6347

**Published:** 2023-04-05

**Authors:** Citra N. Z. Mattar, Jerry K. Y. Chan, Mahesh Choolani

**Affiliations:** ^1^ Yong Loo Lin School of Medicine National University of Singapore Singapore Singapore; ^2^ National University Health Systems Singapore Singapore; ^3^ KK Women's and Children's Hospital Singapore Singapore; ^4^ Duke‐NUS Medical School Singapore Singapore

## Abstract

Proof‐of‐principle disease models have demonstrated the feasibility of an intrauterine gene modification therapy (in utero gene therapy (IUGT)) approach to hereditary diseases as diverse as coagulation disorders, haemoglobinopathies, neurogenetic disorders, congenital metabolic, and pulmonary diseases. Gene addition, which requires the delivery of an integrating or episomal transgene to the target cell nucleus to be transcribed, and gene editing, where the mutation is corrected within the gene of origin, have both been used successfully to increase normal protein production in a bid to reverse or arrest pathology in utero. While most experimental models have employed lentiviral, adenoviral, and adeno‐associated viral vectors engineered to efficiently enter target cells, newer models have also demonstrated the applicability of non‐viral lipid nanoparticles. Amelioration of pathology is dependent primarily on achieving sustained therapeutic transgene expression, silencing of transgene expression, production of neutralising antibodies, the dilutional effect of the recipient's growth on the mass of transduced cells, and the degree of pre‐existing cellular damage. Safety assessment of any IUGT strategy will require long‐term postnatal surveillance of both the fetal recipient and the maternal bystander for cell and genome toxicity, oncogenic potential, immune‐responsiveness, and germline mutation. In this review, we discuss advances in the field and the push toward clinical translation of IUGT.

## Key points

**What's already known about this topic?**

- Transgenic rodent models of monogenic diseases have demonstrated the feasibility and efficacy of intrauterine gene addition therapy.
- Diseases most successfully treated in utero in disease models include haemophilia B, neuronopathic Gaucher disease (nGD), surfactant deficiency syndrome, and certain metabolic diseases.
- Non‐human primate models of haemophilia B have demonstrated long‐term safety and sustained transgene expression.

**What does this review add?**

- Recent experimental models demonstrate promising data using intrauterine nanoparticles and gene editing tools to reverse congenital pathology.

## 1 INTRODUCTION

The Earth's population, currently at 8 billion, is anticipated to increase to 11 billion by the year 2100 despite decelerating population growth over the past decade.^1^ Birth defects incidence will mirror this population growth, particularly in low‐ and middle‐income nations where access to carrier or prenatal screening, genetic diagnostics, or pregnancy interventions is limited, and global migration will increase genetic disease prevalence across high‐income nations as well.^2^ While the incidence of malformations, as well as chromosomal and autosomal dominant anomalies, is predicted to remain stable, the incidence of autosomal recessive diseases will increase as more rare diseases are recognised as genetic syndromes,^3^ and as the rate of fetal diagnosis of monogenic disease increases with prenatal diagnosis.

The life‐time cost of supporting a child with monogenic disease is high. Monogenic diseases affect approximately 1 in 200 pregnancies, account for at least 80% of rare diseases and contribute to ∼39 million disability‐adjusted life‐years (DALYs) in a 2010 estimation (down from >54 million DALYs in 1990), primarily caused by pathophysiological changes initiated during the critical stages of fetal development.^3^, ^4^, ^5^, ^6^ More than 400,000 deaths per year are due to birth defects.^4^ Computational modeling and clinical trial data for genetic diseases, such as severe combined immunodeficiency syndrome (SCID), transfusion‐dependent *β*‐thalassaemia, haemophilia, and spinal muscular atrophy (SMA), demonstrate the substantial health and cost advantages of instituting a cellular or gene modification therapy (GMT) at a younger age and earlier stage of disease^7^, ^8^, ^9^, ^10^; even greater benefits are expected when the affected fetus is diagnosed and treated in utero.

Gene modification therapy encompasses gene editing and gene addition therapy, both modalities of which are in clinical trials for a range of monogenic disorders, and participants may be as young as a few months of age.^11^ A number of these are ideal candidate diseases for in utero therapy due to the detrimental effects of specific protein deficiencies during the critical developmental period. The putative clinical advantages of in utero genetic modification to the affected fetus have been described in numerous reviews.^12^, ^13^, ^14^, ^15^ In essence, greater therapeutic efficacy is possible in the fetus compared to the postnatal recipient, given the greater vector‐to‐mass ratio achieved, the fetal immune system being more tolerant to the administered vector, accessibility through the blood‐brain‐barrier, and the higher concentration of stem cell targets available, increasing the likelihood of reversing or preventing organ pathology when intervening early in the disease timeline. With vector tolerance achieved by in utero administration, it may be possible to repeat this therapy postnatally to boost levels of the desired protein without immune conditioning.^16^, ^17^ In contrast, postnatal therapeutic efficacy has limited impact due to the same factors described, with higher costs for the larger doses required and increased likelihood of a neutralising immune response with age.^18^, ^19^, ^20^, ^21^ Affected children may already have neurological or physical disabilities, and develop growth failure, fertility issues, adverse psychological effects from disease and treatment‐related complications, and early‐onset endocrinological complications. Yet despite the abundance of animal model data showing the therapeutic advantages, clinical trials of fetal GMT have yet to be organized. Here, we review the application of gene modification therapies in utero for monogenic diseases and discuss disease‐specific gene therapy approaches in the fetus to understand clinical efficacy and predict complications.

## 2 DELIVERY OF GENE MODIFICATION TOOLS TO THE FETUS

### 2.1 In vivo and ex vivo administration

The aim of primary in utero GMT is to perform a single prenatal intervention to correct the aberrant genetic function for the life course of the recipient.^15^ This can be achieved with systemic or local administration of the gene therapy tool, via intravenous, intraperitoneal, intraventricular or intracerebral, intratracheal or intraamniotic routes.^22^ Gene delivery strategies are designed to introduce the correct DNA sequence (transgene) to the target cell via integration into the host genome or episomally (outside of the host genome) or to introduce a nuclease that can edit the aberrant gene in situ according to a DNA or RNA template in order to revert it to the wild‐type form.^23^ The gene therapy vehicle can either target the affected organ, such as motor neurons in SMA or Gaucher's disease, or the primary production site of the desired protein, such as the liver for haemophilia. In proof‐of‐principle animal models, fetal gene therapy has proved more successful than postnatal therapy from the perspective of therapeutic efficacy, reversal of pathology, and improvement of clinical outcomes due to the limited degree of tissue damage earlier in the disease timeline, allowing these organs to produce transgenic proteins and to reverse cellular damage more efficiently.^19^, ^21^, ^24^ Younger and less physiologically mature recipient organs are more efficiently transduced and produce greater quantities of transgenic proteins. Further, younger patients have higher disease‐free survival as they have less organ pathology, and undamaged target organs are more receptive to transduction.^25^ This approach offers a valuable alternative option to pregnancy termination or palliative treatment, and fetal therapy can be performed safely without increasing pregnancy loss.^26^

**FIGURE 1:**
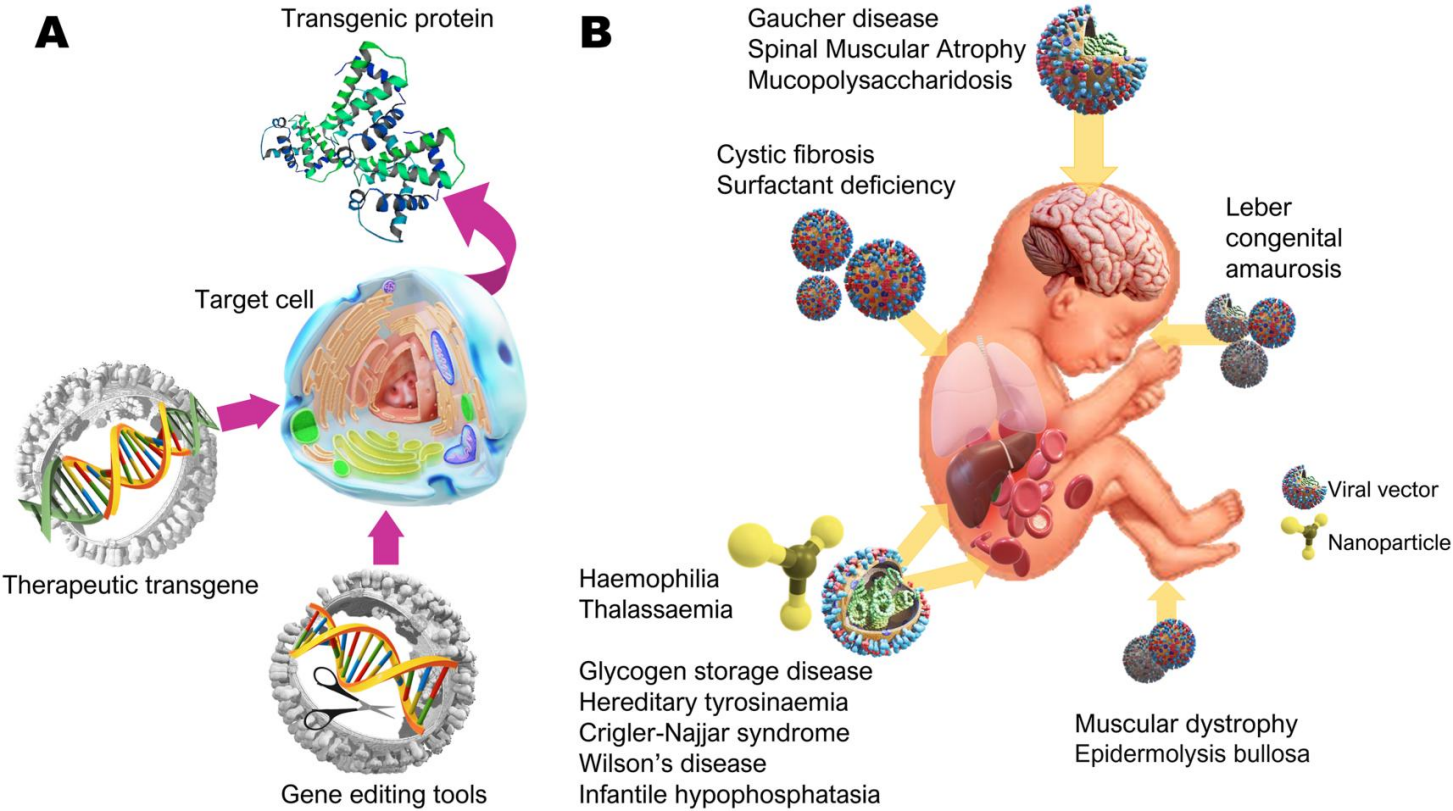
Legend. (A). Gene addition and gene editing therapies use viral or non‐viral vectors to deliver the engineered transgene with cell‐specific or ubiquitous promoters or nuclease and template RNA, respectively. Target cells produce the corrected protein following transduction. (B). Numerous disease models have been partially or completely rescued in utero by targeting specific organs directly (intracerebral, intrahepatic, intraocular, and intra‐amniotic routes) or via systemic delivery (intravenous and intraperitoneal routes).

In utero GMT requires delivery of engineered DNA encoding the desired protein (transgene) packaged in a delivery vehicle (nanoparticle or vector, often of viral origin) designed to display tropism for certain cell types, for example, hepatocytes. The target gene is added to the genome or the host genome is genetically modified, so that the new transgenic protein can be produced by the cell (Figure 1A). This can be performed ex vivo or in vivo, the former requiring the harvest of stem cells from the recipient, genetic manipulation in vitro and autologous transplantation of modified stem cells, the method employed in clinical trials of gene addition or editing for haemoglobinopathies and hereditary immunodeficiencies.^27^ Limited success has already been demonstrated in the context of autologous amniotic fluid‐derived haemopoietic cell transplantation in a sheep model.^28^ Potential advantages of ex vivo approaches are precise correction of target stem cells without affecting non‐target cell types, screening for off‐target mutations in transduced cells, and the ability to expand harvested stem cells after gene modification to increase the yield of corrected cells for transplantation. The downsides are greater material costs, longer preparation time, immune‐conditioning prior to transplantation and the greater risk to the fetus considering that at least two invasive procedures are required.^29^, ^30^

Thus, the in vivo approach would be preferable in utero as this minimises invasive procedures and reduces miscarriage risk. This method is employed in clinical trials of postnatal gene addition therapies using non‐integrating viral vectors targeting the haemophilias and SMA^11^ and has been used effectively to limit pathology in numerous animal disease models of in utero gene therapy (IUGT). Advantages include reduced procedural and material costs and direct delivery of vectors to the target organ, while disadvantages include widespread biodistribution to non‐target organs in the recipient and mother (due to transplacental spread). The in vivo method has been tested in experimental IUGT models. Potential adverse effects of GMT include progressive loss of transgenic protein expression from transduced cells (diluted over time with the recipient's growth),^21^ genotoxicity,^24^ immunotoxicity initiated by the vector or transgene, organotoxicity in target or non‐target organs, and reproductive toxicity with the possibility of gamete transduction.^17^

### 2.2 Gene addition versus gene editing strategies

Gene addition and gene editing are both applicable to IUGT and have been used to target the same diseases (Figure 1A,B). While gene addition introduces the correct segment of DNA into the nucleus so that the cell can produce the desired protein immediately, gene editing corrects the mutation within the native gene.^17^, ^31^ Gene addition places the correct version of the affected gene into the nucleus of the target cell either by integration into the host genome or without integration (the transgene is maintained episomally, outside of the genome), such that sufficient quantities of the correct protein are expressed to reverse the abnormal phenotype.^31^, ^32^ Vectors deliver the transgene coupled with a specific promoter that activates transgenic protein expression only in the target cell, for example, haemopoetic stem cells for haemoglobinopathies. This is particularly important for an in vivo strategy in which several cell types may be transduced by the vector. Production of the correct transgenic protein replaces defective or deficient native protein to correct the clinical pathology. Gene editing relies on molecular scissors to make a double strand DNA break (DSB) at the site of the native mutation. This requires a specific nuclease, donor DNA, and guide RNA to activate homology‐directed repair (HDR) to integrate the new (correct) gene sequence into the genome (knock in). HDR's precision is related to the use of homologous donor DNA acting as a template on which to add the correct sequence of bases. Editing tools induce sequence‐specific DSB (CRISPR‐Cas or Zinc finger nucleases) or single‐strand DNA nicks (base editors), which are repaired by HDR, though non‐homologous end joining, which tends to be error‐prone, often produces heterogeneous insertions and deletions (indels), and acts concurrently with HDR, reducing editing efficacy.^31^

### 2.3 Delivery tools

In vivo delivery is achieved with viral or non‐viral vectors with viral vectors being favored in in utero models because of their superior efficacy.^33^, ^34^ Viral vectors derived from adenoviruses (AdV) and adeno‐associated viruses (AAV) are preferred as gene addition is episomal with a minimal risk of genome integration. Typically regarded as non‐pathogenic vectors, AAV and AdV are associated with immunotoxic reactions which may neutralise transgenic expression, thus diminishing their therapeutic effect, and on the rare occasion this has proven fatal.^35^ Typical adverse outcomes with integrating vectors (onco‐retroviral, gamma‐retroviral, and lentiviral (LV) vectors) include genomic mutagenesis related to obligatory insertion of the transgene into the host genome, which may result in leukemia and other genotoxic events.^24^ Non‐integrating LV vectors are currently in development to overcome this limitation.^36^, ^37^ AdV and integrating vectors are popular vehicles as they can be packaged with large transgenes, while the smaller carrying load of AAV limits its use to packaging shorter transgenes.^29^ The size of the transgene with promoters determines its packaging into viral vectors. Adeno‐associated viruses are deemed the least toxic for in vivo use compared with AdV or LV, but they have the lowest carrying capacity and can only deliver small transgenes (e.g. hFIX). Larger transgenes (e.g. hFVIII or *β*‐globin) can be readily packaged into AdV or LV vectors with larger loading capacities but limited in vivo utility, particularly for in utero use.^31^, ^38^, ^39^, ^40^, ^41^ However, emerging strategies, such as the use of smaller but functional transgenes,^42^ the evolution of a dual AAV system,^43^ or even an all‐in‐one AAV system^44^ have provided solutions for the use of the less immunogenic AAV vectors.

Non‐viral vectors do not present the immunotoxic or genotoxic adverse effects of viral vectors, but tend to be far less efficient at transducing target cells as they lack viral cell entry mechanisms; additionally, most techniques are suitable only for ex vivo cell transduction (e.g. lipofection, nucleofection, and plasmids) though the expanding library of lipid nanoparticles (LNP) designed for programmed release, slow release, and to display specific cell tropism, expands opportunities to use non‐viral technology for in vivo strategies.^45^ Gene therapy vectors are selected for each strategy based on carrying load (larger capacities are required for larger transgenes), tissue tropism (vectors can be designed to show greater affinity for certain cell types), immunogenicity, integration (needed for rapidly dividing stem cells which will lose episomal transgenes with cell division), cell toxicity, and insertional mutagenesis. Editing tools can be packaged in the same way as transgenes but require vectors with larger carrying capacities, such as AdV, LV, or LNP.

Important factors by which to measure the success and safety of the chosen gene modification strategy are transduction efficiency (percentage of transduced cells in the target organ), transgenic protein expression, loss of protein expression with growth, immune‐mediated clearance of transduced cells, off‐target and on‐target integration, mutations and indels, germline mutation, transplacental trafficking of vector, and bystander effects in the mother. We will better understand the applicational benefits and challenges by looking at specific examples and preclinical models. A discussion of in utero stem cell transplantation is beyond the scope of this review.

## 3 HAEMATOLOGICAL DISORDERS

### 3.1 Haemophilias

The most common coagulation disorders are X‐linked haemophilia A (Factor VIII deficiency affecting 1:5000 males) and haemophilia B (Factor IX deficiency affecting 1:35,000 males) and deficiencies of von Willebrand factor (affecting 1% of the population). The haemophilias are ideal conditions to treat in utero, considering the challenges both of severe disease and of conventional treatment.^46^ Severe bleeding diathesis is associated with physiological deficiency of coagulation Factor VIII (FVIII) or IX (FIX), respectively, and is marked by spontaneous bleeding (<1% activity), which improves greatly following a modest increase to 3%–5% activity (mild‐moderate disease and improved quality of life). Factors VIII and IX do not require specific organ expression; though the liver is the main protein factory, ectopic production has not proven detrimental to the treated subject.^46^ Both factors have a wide therapeutic window and supraphysiological expression levels have not demonstrated toxicity in gene therapy recipients.^47^, ^48^ A major adverse effect of recombinant FVIII replacement is inhibitor production in ∼30% of patients. IUGT can induce tolerance to transgenic FVIII and may circumvent inhibitor formation. These factors lower the barrier for effective gene therapy solutions, which when applied in utero can potentially prevent perinatal intraventricular haemorrhage, umbilical cord haematoma, and other birth trauma associated with severe diseases.^49^

Haemophilia A has initially proven a challenge to correct in gene therapy trials despite modest success in murine and canine models in which phenotypic correction has been achieved following systemic administration of AdV, LV, and AAV.^50^, ^51^, ^52^, ^53^, ^54^, ^55^ However, this appears to have been addressed with the development of a functional B‐domain‐deleted human‐factor VIII that can be packaged into AAV5 or AAV3‐subtype 3 vector, demonstrating clinical efficacy.^42^, ^56^ Evidence of IUGT efficacy is limited: a sheep model replicating the spontaneous bleeding phenotype has been described,^57^ but IUGT has demonstrated partial success only in a murine severe haemophilia A model.^58^ Intraperitoneal injection at E15 of the pAdCMVmFVIII AdV vector expressing murine FVIII resulted in FVIII activity at ∼50%–60% of physiological levels at neonatal day 2, declining progressively until no longer detected at day 21. Though experience in correcting haemophilia A in utero is limited, gene marking experiments in preclinical murine, ovine, and non‐human primate (NHP) models have demonstrated that candidate vectors effectively target the liver and produce sustained transgene expression in utero and postnatally.^59^, ^60^, ^61^, ^62^, ^63^, ^64^ Haemophilia B murine models have demonstrated IUGT efficacy using AdV, LV, and AAV, showing that a single prenatal administration of the selected vector produces high levels of FIX in knockout mice without the expression of antibodies and at sufficient levels to normalise defective clotting time.^21^, ^65^, ^66^, ^67^ Importantly, it was demonstrated in this model that quantity and duration of transgene expression were inversely related to recipient maturity, with fetal recipients maintaining long‐term therapeutic FIX levels, outperforming neonatal and adult recipients. Sabatino et al. showed a sustained expression of human hFIX without humoral response in fetal recipient mice following intravenous delivery of AAV1 and AAV2, in contrast to transient hepatocyte transduction and expression in adult and neonatal recipients due to neutralising antibodies to AAV. Using the same IUGT model treated with LV‐hFIX, Waddington et al. achieved phenotype correction without a humoral response.^21^, ^65^ Addressing a deficiency of von Willebrand factor, a mouse model of A disintegrin and metalloprotease with thrombospondin 13 (ADAMTS13) deficiency (a cause of congenital thrombotic thrombocytopenic purpura) was corrected with intravenously delivered LV‐ADAMTS13, achieving sustained ADAMTS13 expression and correction of prothrombotic phenotypes.^68^

Sheep and NHP preclinical IUGT models have permitted us to learn about long‐term outcomes of IUGT despite the lack of transgenic models to demonstrate phenotype correction. Ovine fetuses treated at 0.3 and 0.7 gestational age (GA) with intravenous AdV‐hFIX expressed moderate to high hFIX plasma levels, with a few treated lambs showing anti‐adenoviral and anti‐hFIX antibodies.^69^, ^70^ Most robust transduction was observed in the liver with weaker transduction of other intraperitoneal viscera, and transgene expression was exclusive to hepatocytes; a small number of procedure‐related perinatal deaths occurred. Intraperitoneal delivery of Moloney murine leukemia retrovirus encoding the bacterial *neo* reporter gene at 0.3–0.4 GA confirmed 1 transduced sperm in 6250, several log‐fold below naturally occurring insertional mutations of 1:8 in humans, and lower than the 1:6000 sperm threshold accepted by the FDA.^62^, ^71^

We used a NHP model to demonstrate the clinical translatability of this approach, achieving supraphysiological hFIX expression in NHP recipients using AAV8 and AAV5, both of which are liver‐tropic,^16^, ^17^, ^72^ delivered via intravenous, intraperitoneal and intracardiac routes with high perinatal survival of injected fetuses. Importantly, we demonstrated the feasibility of a single intrauterine dose, given that transgene expression was maintained in most animals for several years with minimal neutralising response to transgene or AAV, and minimal immunotoxicity at the doses used. Through multi‐year surveillance of IUGT NHP, we demonstrated that transgene expression is related to serotype, GA, and gender of recipients.^17^, ^72^ Human FIX and FX were both expressed at therapeutic levels using the same AAV vectors, which were maintained over at least 50 months, despite rapid infant growth.^16^ Subtherapeutic expression was associated with transient neutralising antibodies to AAV. Postnatally, IUT recipients with subtherapeutic expression challenged with the same AAV without immunosuppression showed improved transgene expression despite transient humoral and cellular reactions. Transient transaminitis occurred with AAV5 but none of the affected infants developed clinical sequelae. Low‐level AAV integration was observed at random sites in host genome.^16^, ^72^ AAV particles were not isolated from purified oocytes of female IUT recipients, while transplacental trafficking of AAV produced a mildly positive humoral response in mothers. This model was thus able to demonstrate the safety and feasibility of IUGT, critical factors for therapeutic outcomes, and the degree to which adverse effects may occur, outcomes that are informative for strategic approaches to other monogenic diseases.

### 3.2 Haemoglobinopathies

The haemoglobinopathies are the most common monogenic condition globally and collectively impose a tremendous burden on medico‐economic resources particularly in low‐ and middle‐income nations. Approximately 7% of the world population are carriers and this incidence may continue to rise with population migration trends.^73^, ^74^, ^75^ Currently, 300,000–500,000 severely affected children are born annually. Curative haemopoetic stem cell transplantation (HSCT) for transfusion‐dependent haemoglobinopathies requires matched HSC with full myeloablation to create the bone marrow niche for donor cell engraftment, the outcomes of which are directly related to the recipient's youth, health, and absence of splenomegaly, hepatic fibrosis, and iron deposition. Children with pre‐transplantation risk factors have a lower survival rate (60%), higher graft‐vs‐host disease (5%–8%), and graft rejection (∼30%–40% of recipients with mixed chimerism), following HSCT.^10^, ^76^, ^77^ The 25%–30% of patients who do not have a suitable donor require lifelong blood transfusions iron chelation, risk growth failure, and endocrinopathies from end‐organ iron deposition.^78^, ^79^ GMT offers a potential cure by editing or replacing the mutant gene. Current clinical trials use an ex vivo approach to either edit the native *β*‐globin gene (Vertex/Sangamo) or integrate the correct *β*‐globin into the host genome (Telethon/Bluebird Bio).^40^, ^80^, ^81^, ^82^, ^83^ Harvested HSC are genetically modified and examined for off‐target effects prior to autologous transplantation.^80^, ^84^ This is less favourable than an in vivo approach due to the need for two invasive procedures required to obtain and retransplant HSC. Given the prohibitive lifetime costs of lifelong transfusions with iron chelation^85^ and of autologous HSCT,^86^ and the benefits of treating younger and smaller patients, there is a strong cost‐to‐benefit incentive to pursue fetal treatment.

IUGT has been investigated in knockout murine models of major *α*‐ and *β*‐thalassaemias using LV to integrate the respective globin transgenes into HSC. Han et al. delivered a single intravitelline vein injection of the dANS9‐cppt‐ha LV vector expressing human α‐globin driven by the *β*‐LCR (locus control region) and *β*‐globin gene promoters into the *α*‐thalassaemia mouse at E14.5,^87^ which produced erythroid‐specific transgene expression.^88^ α‐globin levels in liver, spleen, and peripheral blood peaked at 20% of mouse α‐globin levels by 130 days, but eventually declined to <5% by 7 months. Low expression in erythroid colonies was postulated to be due to transgene silencing or suboptimal targeting of HSC. Two studies reported the efficacy of direct in vivo IUGT in *β*‐thalassaemia mice using the *β globin*‐expressing clinical vector GLOBE‐LV, in which erythrocyte‐specific *β‐globin* expression is driven by the *β*‐LCR^89^ and which is now in phase I/II clinical trial (NCT02453477); genetic modification of 15%–30% of maturing erythroblasts was sufficient for correction.^90^ Dighe et al. delivered GLOBE‐LV intravenously via the vitelline vein at E15‐16 into the Hbb^Th3/+^ knock‐in model of severe *β*‐thalassaemia intermedia, which exhibits splenomegaly and typical peripheral blood microcytosis and anisocytosis.^91^ Treated recipients had detectable vectors in peripheral blood and bone marrow mononuclear cells and in the liver (<1 vector copy/cell) for up to 20 postnatal weeks. Mean corpuscular volume and erythrocyte counts improved significantly though they remained below the haematological indices in wild‐type mice. Shangaris et al. performed LV‐IUGT using the Humanised CA mouse model of severe *β*‐thalassemia major, which exhibits splenomegaly, impaired cardiac function, and extramedullary haemopoesis.^92^ Globe‐LV was injected intrahepatically at E13.5 achieving <0.1 vector copy/cell. Haemoglobin concentration of treated heterozygous pups was similar to non‐thalassemia controls, but extramedullary haemopoesis and iron deposition in spleens were reduced and cardiac function improved; these effects became less pronounced with age suggesting transcriptional silencing. Integration sites were identified in the liver and bone marrow. Both the studies of in utero LV‐mediated GMT demonstrated largely beneficial effects, but leukemogenic risk from vector insertion is present particularly in rapidly dividing HSC, similar to retroviral GMT for hereditary immunodeficiencies.^93^, ^94^

A recent demonstration of intravenous and intraamniotic delivery at E15.5‐E16.5 of biodegradable polymeric nanoparticles carrying triplex‐forming peptide nucleic acids and single‐stranded donor DNA^95^ illustrated widespread nanoparticle biodistribution, concentrated in fetal liver (without trafficking to maternal liver), correcting the pathological mutation in human *β‐globin* in the transgenic Hbb^Th4/‐^ thalassaemia mouse, leading to sustained postnatal elevation of haemoglobin, reversal of splenomegaly, and improved survival following a single in utero dose. Editing efficacy was ∼6% with undetectable off‐target mutations in bone marrow. Persistent correction of anemia suggested that fetal HSC were effectively targeted, and phenotype correction was achieved with <10% editing frequency.

## 4 NEURODEGENERATIVE DISEASE

Neurogenetic disorders may manifest prenatally causing extensive and early‐onset brain injury; examples include the mucopolysaccharidoses (MPS) and Gaucher type‐II (acute infantile neuronopathic disease). Postnatal enzyme replacement efficacy is limited by the increasingly impermeable blood‐brain barrier and irrecoverable neuronal damage.^96^ An effective and safe gene therapy that can correct the underlying mutation, prevent early‐onset neuropathy, and preserve the developing brain is urgently needed.^97^ These conditions are ideally treated in utero. Fetal brain and spinal cord degeneration were prevented in a mouse model of MPS type VII (Sly syndrome), a lysosomal storage disease causing growth retardation, skeletal abnormalities and intellectual disability, with a single dose of AAV1‐HβH transferring the human *β*‐glucuronidase (*GUSB*) promoter and cDNA, administered at E15.5 during active neurogenesis.^98^ Widespread symmetrical CNS transduction was observed resulting in therapeutic *GUSB* activity levels throughout the cortex, cerebellum, hippocampus, and brainstem. This prevented neurodegeneration throughout the brain and spinal cord. Small amounts of *GUSB* enzymatic activity were detected in visceral organs of treated mice.

The Idua‐W392X MPS‐IH mouse model recapitulates phenotypic features of MPS type I (Hurler syndrome, MPS‐IH) commonly caused by the *IDUA* G→A (W402X) mutation in humans. Split‐intein AAV9 (a dual AAV strategy) was used to deliver the adenine base editor and guide RNA targeting the *Idua* G→A (W392X) mutation to Idua‐W392X murine fetuses intravenously at E15.5.^99^ Prenatal treatment resulted in high editing efficiencies (around 12%) in liver LGR5+ progenitor cells and cardiac cells, including myocytes persisting to 6 postnatal months, reduced urine glycosaminoglycans, 100% survival (vs. 40% in untreated mice), reduced skull and femur cortical bone deposition, and no germline editing. There was no off‐target editing above background spontaneous mutation frequency and low indel (insertion/deletion) rates.

We have demonstrated in fetal murine and NHP models that AAV9 can effectively target neuronal cells when injected directly into the cerebral ventricles or intravenously.^18^, ^100^ AAV9 efficiently transduced neurons, astrocytes, oligodendrocytes, dorsal root ganglia, and retinal layers in the central (CNS) and peripheral (PNS) nervous systems,^100^ and additionally, transduced epithelial cell layers in the gastrointestinal, renal, and respiratory systems.^18^ This prompted the study of AAV9‐mediated delivery of a functional glucocerebrosidase transgene (*GCase*) to a mouse model of nGD,^19^ which is caused by *GBA1* mutations encoding for *β*‐glucocerebrosidase, an enzyme associated with lysosomal and cell membranes, and resulting in impaired *β*‐glucocerebrosidase activity and neurotoxicity from glucocerebroside accumulation in macrophages and microglia,^101^, ^102^, ^103^ causing progressive neuroinflammation, neuronal loss, cerebellar ataxia, dementia, epilepsy, and paralysis. The acute childhood form of nGD is lethal and untreatable as exogenous *β*‐glucocerebrosidase cannot cross the blood–brain barrier.^104^ Massaro et al. successfully rescued the fetal murine nGD model with a single intracranial injection of human AAV9‐GBA at E16, restoring neuronal glucocerebrosidase expression.^19^ While untreated knockout mice exhibited fatal neurodegeneration and severely truncated lifespan, fetal IUGT recipients survived until at least 18 postnatal weeks, showed normal non‐spasmodic mobility, and brain distribution of microglia, astrocytes, and neuronal lysosomes was restored. While the use of IUGT in enzyme deficiency disorders has yet to enter clinical trials, an interesting similar approach uses in‐utero enzyme replacement therapy to treat Pompe's Disease, another enzyme deficiency disease, which has led to improved phenotype in utero and better outcomes at 13 months of life.^105^

Another example of a life‐threatening neurodegenerative disease amenable to IUGT is SMA, caused by mutation or deletion of the survival motor neuron 1 gene (*SMN1*) on chromosome 5q13, resulting in the depletion of the ubiquitous SMN protein required for multiple fundamental cellular homeostatic and bioenergetic pathways.^106^ This affects 8–10/100,000 newborns globally per annum, and enhanced neuronal death is already detectable in utero.^107^, ^108^ Intracerebroventricular AAV9 delivering the human *SMN* to peripheral motor neurons in a murine knockout model of SMA extensively transduced various neuronal areas of the CNS with a great quantity of these being neural stem cells.^109^ Clinical correction of muscular atrophy and neurological symptoms, with increased lifespan and improved postnatal growth, was observed in fetal IUGT mice. Intraperitoneal injection resulted in more widespread and less CNS‐concentrated cellular transduction, and SMN transgenic protein expression was higher in the brain and spinal cord than in wild‐type mice. This therapy has now been developed by a biotech company into an AAV vector sold commercially as Zolgensma®, but at a cost of €2 million even for a small child, the medication is out of reach for many families in need.^110^ These experimental models give us a powerful demonstration of the potential benefits achievable with a single IUGT dose, particularly in a perinatally lethal or devastating condition without effective postnatal replacement therapy.

## 5 CONGENITAL PULMONARY DISEASE

IUGT has successfully targeted monogenic pulmonary diseases, including congenital surfactant deficiency and cystic fibrosis. Intraamniotic delivery of GMT tools is ideal to target the developing lungs, primarily the pulmonary epithelial cells.^69^, ^111^, ^112^ This will be a useful strategy for cystic fibrosis and inherited surfactant protein syndromes caused by mutations in surfactant system genes (*SFTPB*, *SFTPC*, or *ABCA3*), resulting in respiratory failure at birth, perinatal death, or chronic diffuse lung disease.^113^ Cystic fibrosis is a multisystem disease caused by exonic and intronic mutations in the cystic fibrosis transmembrane regulator *CFTR* gene, resulting in the dysregulation of cAMP‐regulated chloride channels controlling salt and fluid homeostasis in epithelial cells; this produces viscid secretions and epithelial cell dysfunction and ultimately mortality from respiratory failure.^114^ Alleviating respiratory disease may substantially improve quality of life and decreased mortality. Efficient system‐wide transduction of epithelial cells has been shown with fetal administration of AAV9, AdV, and LV vectors.^18^, ^112^, ^115^ A ferret model CF bearing homozygous *CFTR* ^*G551D/G551D*^ mutations has demonstrated the utility of in utero treatment. Administration of ivacaftor (a CFTR potentiator) in utero via maternal ingestion produced partial improvement of epithelial cell dysfunction (in pancreas, intestine, and male reproductive tract), improved pancreatic exocrine and islet cell function, reduced pulmonary secretions and bacterial infections, and accelerated growth and survival, while withdrawal of treatment at any time accelerated pathology and morbidity. This model demonstrates the importance of epithelial function during fetal development and the magnitude of benefit that might be achievable with an IUGT approach.^116^ However, GMT strategies have not been as successful. Buckley et al. injected the knockout *Cftr* ^*tmlCam*^ CF mouse model intra‐amniotically with an AdV vector AdCFTR. Though both AdV DNA and transgenic CFTR expressions were detected postnatally in the gut and lungs of treated animals, there was no significant survival advantage over sham‐injected control animals.^111^

Surfactant, produced by alveolar type II (AT2) cells and critical for the structural integrity and function of alveoli, maintains low alveolar surface tension for efficient gaseous exchange. *Sftpc* ^*I73T*^ mutations cause intracellular accumulation of SP‐CI73 T proprotein, resulting in AT2 cell injury, hypertrophy, and arrest of lung morphogenesis. A knockin mouse model with the human *SFTPC* ^*I73T*^ mutation develops perinatally lethal severe diffused parenchymal lung damage in utero and may potentially be rescued with fetal gene editing. Alapati et al. successfully transduced AT2 cells in the *R26* ^*mTmG/+*^ reporter mouse using AdV‐eGFP vectors injected at E14 with inhalation into fetal lungs enhanced by concurrent administration of theophylline and 10% CO_2_. This allowed for AT2 rescue in the *Sftpc* ^*I73T*^ knockin mouse, characterised by fibrotic lung disease secondary to intracellular accumulation of mutant proprotein and perinatally lethal homozygous fetuses with arrested pulmonary development, by excision of the mutant *Sftpc* ^*I73T*^ from hybrid *Sftpc* ^*I73T*^/wild‐type fetuses and editing in *Sftpc*, correcting AT2 cell function and converting the phenotype from a lethal to non‐lethal one. The *Sftpc* gene‐edited band was detected in the lung tissue of Ad.Sftpc.GFP‐injected mice but not in controls. Treated recipients showed improved lung morphology, reduced severity of the lethal interstitial lung disease, and increased survival compared to untreated mice. Alveolarization approximated normal lungs in wild‐type mice and gene editing only occurred in AT2 cells.

Gene therapy targeting the lung is challenging due to mucus secretion, chronic inflammation, and high turnover of airway epithelium. These models show the promise for in utero targeting of the fetal lung for conditions that are difficult to treat postnatally in the damaged lung. The numerous postnatal clinical trials show limited benefits for CF gene therapy^117^; the more successful gene editing strategies can be adapted for fetal application, and benefits are anticipated to be greater than expected in adult recipients.

## 6 METABOLIC DISEASES

The liver is the producer of multiple proteins. Often, loss‐of‐function mutations cause aberrant protein formation or protein deficiency, resulting in abnormal metabolic processes, which may even manifest in utero, such as the lysosomal storage diseases.^118^ Occasionally, loss‐of‐function mutations can benefit the individual, particularly for a disease caused by pathological accumulation of metabolites, such as in hereditary tyrosinaemia (HT1), a severe childhood disease characterised by accumulation of tyrosine and its byproducts in the liver, kidneys, and CNS causing organ failure and mental retardation.^119^ Conventional treatment includes avoidance of tyrosine‐containing foods and nitisinone, a hydroxyphenylpyruvate dioxygenase (HDP) inhibitor.^120^ Early onset pathology and the urgency to initiate nitisinone treatment in the neonate increase the drive toward an appropriate intrauterine therapy. Rossidis et al. demonstrated the feasibility of liver‐targeted GMT to reverse the pathology of HT1 in a murine model.^121^ The Fah knockout mouse carries a deficiency of fumarylacetoacetate hydrolase (*FAH*) resulting in tyrosinemia. *Fah−/−* homozygous mutants manifest the lethal phenotype of HT1 and require treatment with nitisinone to inhibit HDP and thus tyrosine accumulation.^122^ *Hpd* is upstream to *Fah* in the tyrosine pathway, and mice carrying an *Hpd* mutation will manifest a much less severe phenotype. This strategy of phenotypic rescue by way of introducing an in vivo suppressor mutation has been demonstrated in the adult murine model.^122^ In utero delivery of Ad‐BE3‐Hpd (packaging Base Editor 3) at E16 introduced a nonsense loss‐of‐function mutation into *Hpd* and treated *Fah−/−* fetuses exhibited significantly lower transaminases and total bilirubin, and greater weight gain compared to sham‐treated fetuses. Editing rate was ∼15% exclusively in liver at 2 weeks of age; edited cells demonstrated survival advantage and expansion, and off‐target mutations and indels were infrequent. Treated pups showed 89% survival at 3 postnatal months.^121^ This strategy can be applied to other inborn errors of metabolism that will benefit from loss‐of‐function mutations, such as familial hypercholesterolaemia, which may be ameliorated by reducing the function of the *PCSK9* gene.^121^

Several metabolic disorders have been the target of various IUGT strategies in proof‐of‐principle animal models. Crigler–Najjar Type I (uridine diphosphate glucuronosyl transferase 1A1 deficiency) causes perinatal liver failure and severe encephalopathy.^123^ Severe Type I disease was partially corrected in the Gunn rat model, and the clinical phenotype improved to a benign Type II disease with intrahepatic injection of the PGKUGT1A1 LV vector expressing human *glucuronosyl transferase 1A1*, resulting in a 45% decrease in serum bilirubin.^124^ Wilson's disease, characterised by aberrant copper transport and progressive hepatic and neurological disease caused by *Atp7b* mutation, was successfully reversed in *Atp7b−/−* knockout murine fetuses with LV vector LSP.GFP.huATP7B delivered intracardiac at E10, resulting in increased ATP7B expression, decreased hepatic copper content, and restoration of normal liver morphology.^125^ Treated pups had similar transaminase and bilirubin levels as wild‐type mice at 4 postnatal weeks. The *Akp2−/−* model mice carries the phenotype of severe human infantile hypophosphatasia (HPP), exhibiting fetal skeletal dysplasia, growth and respiratory failure, bone hypomineralization, seizures and death by two postnatal weeks caused by *ALPL* mutations. Intraperitoneal injection of AAV9‐TNALPD10 (AAV9 expressing bone‐targeted tissue‐nonspecific alkaline phosphatase) at E15 resulted in pups with normal growth, elevated alkaline phosphatase and mineralisation in bone, and improved seizure‐free survival up to 8 weeks.^126^

## 7 OTHER MODELS OF IN UTERO GENE THERAPY

Hereditary blindness caused by Leber congenital amaurosis, a condition of *RPE65* mutation reducing RPE65 expression in the retinal pigment epithelium and causing severely abnormal retinal function, was reversed following intraocular injection of AAV1 and AAV2 in utero in *Rpe65−/−* mice at E14.^127^ A single subretinal injection of AAV2/1‐CMVhRPE65 allowed substantial recovery of rhodopsin and greater photoresponse sensitivity in treated eyes. The *mdx* mouse model of Duchenne muscular dystrophy (DMD), a progressive muscle degeneration most commonly caused by dystrophin gene frameshift mutations, is characterised by a single point mutation in exon 23 of the dystrophin gene, resulting in the deficiency of dystrophin expression in muscle.^128^ DMD phenotype in *mdx* mice was partially corrected by in utero intramuscular injections of high‐capacity AdV^129^ and intraperitoneal injections of AAV8^130^ at E16, both vectors expressing the minidystrophin gene, restoring dystrophin‐associated glycoprotein complex in muscle fibers with improved histology and functionality, albeit with low expression levels following AdV‐IUGT due to anti‐AdV antibodies. Single‐strand oligodeoxynucleotides (ssODNs), designed to target the splice donor site of exon 23 downstream of the mutation, induce a single‐base alteration at the splice junction resulting in excision of exon 23 during mRNA splicing with reading frame restoration of the dystrophin protein.^131^ In utero intramuscular injection of ssODNs into the hind limbs of fetal *mdx* mice at E16 produced dystrophin expression in skeletal muscle fiber, which was stable over 4 postnatal months,^132^ demonstrating the usefulness of ssODNs for fetal therapy as they diffuse efficiently in skeletal muscle, carry low toxicity, and correct actively dividing cells to restore full‐length dystrophin protein expression. Herlitz junctional epidermolysis bullosa initiates perinatally lethal severe and extensive mucocutaneous skin blistering and is caused by genetic mutations affecting laminin‐α3, laminin‐β3, or laminin‐χ2 polypeptides; the phenotype is reproduced in the homozygous LAMB3IAP mouse, which completely lacks laminin. Intra‐amniotic delivery of LV encoding *laminin‐β3* at E8 facilitated the long‐term expression of laminin‐332 in the skin and mucosal basement membrane, correcting skin pathology and restoring ∼60% hemidesmosomal structures, although treated pups still died within 48h of birth.^133^

## 8 DISCUSSION

Gene addition and gene editing are powerful tools and can often be applied to the same diseases. They each have unique limitations, and strategies for various diseases will require individualisation and optimisation.^23^, ^40^, ^134^, ^135^ Gene addition therapy requires a precise vector product with the appropriate promoters, and vector design strongly affects transduction efficacy. The challenges with integrated or episomally expressed transgenes are the control of expression, particularly in stem cells that are more susceptible to vector‐induced apoptosis following gene addition and promoters that are prone to methylation and silencing, producing variable, inconsistent, or inappropriate expression. In animal studies, gene addition therapy has often shown greater efficacy than gene editing.

Gene editing is attractive because without the addition of transgenic DNA, the perceived risks related to disruption of the genome or transcription of transgenic DNA are minimal. Editing can potentially correct multiple mutations within the gene of interest allowing one specific tool to be applied to different diseases originating from the same segment of DNA. Gene editing leaves the cell's transcription mechanisms and regulatory elements intact while precisely correcting the specific mutation in the gene's native environment. Thus, natural regulatory elements, including switching the essential gene on or off at the appropriate times, are maintained and continue to ensure physiological protein expression without affecting critical cell signaling or activation. Diseases characterised by aberrant physiological processes (e.g. cell activation and intracellular signaling) require correction of genes that govern temporal and physiologic control, which is more difficult to correct by gene addition as vector design may not completely replicate the regulatory sequences associated with the mutant gene within the space constraints of the viral vector. Here, it would be more feasible to correct the mutation in situ without disturbing the regulatory elements, so that the corrected gene can function within its normal expression patterns.

It is generally agreed that the high expenses and associated risks of GMT can be mitigated by a three‐pronged approach: treating patients at a younger age and in better physical health, increasing the number of patients receiving therapy, and using the in vivo approach preferentially,^110^, ^136^ all of which are represented in an intentional IUGT strategy. There should be an international consensus on the highest priority diseases to target, which may vary geographically, as gene therapy products are expensive to produce and require lengthy validation of efficacy and safety according to good manufacturing practice regulations.^13^, ^137^ Numerous factors influence the decision to proceed to clinical applications, such as population carrier frequency, affordability of conventional medical care as well as the priority placed on advanced therapeutics, and ethical considerations will associate closely with societal acceptance of genetic diseases, pregnancy termination, and pregnancy interventions. Focusing research and clinical efforts on the more common diseases like the haemoglobinopathies, SMA, and cystic fibrosis may increase the likelihood of reaching global clinical trials of IUGT in the near future. Unresolved issues need to be addressed through improving vector design and longitudinal surveillance in large animal models, and these include long‐term disruptive effects of vector integration, genotoxicity and malignant change, future reproductive health, and long‐term maternal off‐target effects. The time to step into clinical trials is fast approaching, and fetal medicine providers and gene therapists should function within consensus‐lead guidelines to carefully, but boldly, step into this new therapy.

## CONFLICT OF INTEREST STATEMENT

The authors declare no financial conflict of interest.

## Data Availability

Research data are not shared.
